# A case report of central core disease with repeated foaming at the mouth as the initial symptom

**DOI:** 10.1097/MD.0000000000036332

**Published:** 2023-12-01

**Authors:** Qiu-yue Zhang, Yang-yan Yin, Lu Bai, Xuan Xu

**Affiliations:** a Department of Pediatrics, the First Affiliated Hospital of Hainan Medical College, Haikou, China.

**Keywords:** case report, central core disease, diagnosis, foaming at mouth, genes, hypotonia

## Abstract

**Background:**

Central core disease (CCD) is a congenital myopathy primarily observed in infants and children. It frequently manifests as limb weakness or delayed motor development, characterized by gradually progressing or non-worsening weakness and muscle atrophy primarily affecting the proximal limbs. Joint deformity is a prevalent clinical feature. Presently, there is no targeted treatment available for this condition.

**Case description:**

The infant, who was 42 days old, showed a repeated occurrence of foaming at the mouth for more than a month as the initial symptom. Initially, the local clinic misdiagnosed it as softening of the thyroid cartilage. However, when the infant underwent bronchoscopy at our hospital, it was discovered that the pharyngeal muscle was loose, and there was noticeable retraction of the base of the tongue. Additionally, the infant displayed evident hypotonia and an increase in creatine kinase levels. By conducting a thorough genetic examination, we confirmed that the infant had CCD.

**Conclusion:**

The onset of CCD may manifest as various symptoms. Medical practitioners need to be attentive in recognizing individuals who experience recurring pneumonia along with reduced muscle tone during the course of clinical diagnosis and treatment.

## 1. Introduction

Central core disease (CCD) is a rare inherited muscle disorder that was initially documented by Shy and Magee in 1956 and officially named by Greenfield in 1958.^[1]^ It is characterized by a distinctive pathological feature where a single clear core structure appears at the central position of type I muscle fibers, visible through staining with phosphorylase and oxidase. Type I fibers are primarily affected, without any fiber necrosis or proliferation. The core structure extends throughout the entire length of the muscle fiber in longitudinal sections.^[2]^ The incidence of this disease is low, occurring in only 1 per 100,000 live births. Clinical symptoms mainly involve non-progressive or slowly progressive weakness in the muscles of the trunk and proximal areas. Patients often encounter orthopedic problems such as congenital hip dislocation, spinal curvature, and clubfoot deformity.^[3,4]^ Some individuals experience severe muscle weakness from infancy, while others develop symptoms later in adulthood, suggesting significant variation in clinical presentation, likely influenced by the severity of the genetic mutation.^[5]^ Advanced sequencing techniques have proven highly effective in identifying RYR1 mutations in patients with CCD.^[6]^

## 2. Clinical material

Chen, a 42-day-old male infant, was brought to our hospital on March 1, 2023. He had been experiencing repeated episodes of foaming at the mouth for more than a month and had also been having difficulty breathing for over 3 weeks.

### 2.1. Medical history

The infant did not show any obvious reasons for experiencing foaming at the mouth or symptoms like fever, cough, and shortness of breath more than a month before being admitted to the hospital (5 days after birth). At the onset of the illness, the infant did not receive any diagnosis or treatment. However, 3 weeks ago, the infant started experiencing shortness of breath with a bluish tint around the mouth. The infant was then admitted to Sanya People Hospital in Hainan Province, where he was diagnosed with several conditions, including neonatal pneumonia, type I respiratory failure, softening of the thyroid cartilage, neonatal hyperbilirubinemia, and mild anemia.

Various treatments were administered, such as noninvasive ventilator support, ceftazidime for infection, nebulization, blue light therapy for jaundice, myocardial nutrition, and vitamin D supplementation. Unfortunately, these treatments did not significantly improve the condition of the infant compared to previously. Throughout this time, the infant also experienced recurring abdominal distension. Upon the request of the family, the infant was transferred to our emergency department and admitted to the pediatric intensive care unit with a diagnosis of “severe pneumonia,” for further inpatient treatment. The mental state and sleep patterns of the infant were normal. He was receiving oral feeding but with reduced milk intake (from 90 mL every 3 hours to 70 mL every 3 hours), exhibited regular bowel movements, and showed poor weight gain (birth weight of 3.3 kg, admission weight of 4.1 kg). Personal history: The infant was conceived via in vitro fertilization. The mother had an obstetric history of Gravida 3, Para 3. The infant was born at full-term through a cesarean section as the mother had scarring in the uterus. There were no instances of birth asphyxia, and there was no evidence of birth trauma. Following birth, a combination of breast milk and formula feeding was predominantly utilized, and the infant received the initial doses of Bacillus Calmette-Guerin and hepatitis B vaccines. Family history: The father, who is 43 years old, had a medical history of glycosuria. The mother, aged 36, was in good health. The infant has 2 sisters, aged 14 and 11, who are experiencing typical growth and development without any issues.

### 2.2. Physical examination upon admission

T: 36.5°C, P: 138 times/min, R: 49 times/min, BP: 93/46 mm Hg, WT: 4.1 kg. The entire body showed scattered congestive rash. The rash faded when pressed and then swelled again. The largest rash, measuring approximately 3 cm × 3 cm, was located on the trunk. There were no visible signs of jaundice or bruising on the remaining skin. The superficial lymph nodes throughout the body were unaffected. The lips appeared red, and there were no noticeable abnormalities in the oral mucosa or tongue. The pharyngeal mucosa was congested, and the infant had a thin and weak cry, as well as slight shortness of breath. There was a visible depression in the lower sternum, known as the triple concave sign, which became more pronounced during inhalation. Both lungs had thick respiratory sounds accompanied by audible wet rales. There was no abnormal bulging in the chest area, there was normal apex heartbeat, and the cardiac margin was small. The heart rhythm was regular, with a heart rate of 138 beats/min, and no murmurs or pericardial fricative sounds were detected. The abdomen was distended but soft and the liver could be touched at 1 cm below the costal margin with fair mobility. There were no noticeable palpable masses under the rib cage on the left side, and normal bowel sounds were present. The infant had decreased muscle strength and tension in all 4 limbs, a weak holding reflex, slightly weak sucking reflex, poor head control, and no signs of meningeal irritation.

### 2.3. Auxiliary examinations

Results of blood, urine and stool tests were normal. The erythrocyte sedimentation rate was within the normal range, and the levels of procalcitonin, IL-6, and C-reactive protein were also found to be normal. Respiratory tract pathogens: Influenza A antigen positive (+), 2019-nCoV nucleic acid negative (−).

The bronchoalveolar lavage fluid was negative for pathogens and blood cultures were negative.

Culture and identification of bacteria and fungi in phlegm: Moraxella catarrhalis (Branhan) is sensitive to amoxicillin/clavulanic acid (S), cefuroxime (S), azithromycin (S), ceftriaxone (S), erythromycin (S), etc. Upon conducting a reexamination after a period of 2 days, the results were negative.

Biochemistry: Creatine kinase 3015 U/L↑; Creatine kinase isoenzyme 176.4 U/L↑; Aspartate aminotransferase 112 U/L↑;. Blood gas: PH: 7.327↓; Alveolar-arterial oxygen partial pressure: 1.85 KPa↑; Free calcium concentration: 1.33 mmol/L↑; Carbon dioxide partial pressure: 8.16 KPa↑; Actual bicarbonate: 31.3 mmol/L↑; Residual base: 3.5 mmol/L↑ and total carbon dioxide: 33.2 mmol/L↑; Normal level of lactic acid; Plasma ammonia 46 μmol/L↑.

No obvious abnormality was found in thyroid function test and the electrocardiograph was normal.

Ultrasound examination: The anterior cardiac septum showed a slight reduction in movement. The measurements of left ventricular function were within the normal range. The closure of the oval fossa had not yet occurred. There was no presence of fluid in the pericardial cavity. There were no apparent abnormalities found in the liver, gallbladder, spleen, or pancreas. A small quantity of fluid was detected in the abdominal cavity. No obvious abnormalities were found in the abdominal bowel. An abnormal echo was observed in the bladder, indicating possible bladder deposits. No obvious abnormalities were found in both kidneys or the bilateral ureters. Computerized tomography (CT) scan: Upon admission, 3-dimensional spiral CT scanning revealed inflammation in both lungs. The airway appeared normal without any apparent abnormalities. Subsequent imaging after a period of 10 days indicated a slight reduction and absorption of multiple inflammations in both lungs. Magnetic resonance imaging: The cranial magnetic resonance imaging and diffusion weighted imaging did not reveal any obvious abnormalities.

Electronic bronchoscopy: 1. Pharyngeal relaxation; 2. Retracted tongue root; 3. Tracheobronchial endometritis; 4. Gastroesophageal reflux.

### 2.4. Preliminary diagnosis at admission

1. Severe pneumonia; 2. Influenza A virus infection; 3. Type II respiratory failure.

### 2.5. Diagnosis and treatment process

Following admission, the infant experienced difficulties with feeding and swallowing. Additionally, the infant encountered 2 instances of intermittent cyanosis within a 30-minute timeframe after oral feeding. However, this cyanosis was alleviated through plantar stimulation. Consequently, the decision was made to switch to nasal feeding. The infant underwent electrocardiograph monitoring and received oxygen inhalation through a nasal catheter. Noninvasive ventilator-assisted ventilation was also employed. To combat infection, the infant was administered cefoperazone sodium, sulbactam sodium, and oseltamivir phosphate particles. Ambroxol hydrochloride and acetylcysteine were utilized to help eliminate phlegm. Creatine phosphate sodium and coenzyme Q10 were administered to nourish the myocardium. Glutathione and bifendate dripping pills were used for liver protection. Additionally, group B vitamins and levocarnitine were included in the treatment. Aluminum phosphate gel was employed to neutralize gastric acid and reduce reflux. Intravenous nutrition was administered to provide symptomatic support treatment and enhance relevant examinations. After undergoing the mentioned treatment, the foaming at the mouth and the appearance of the triple concave sign showed improvement in comparison to the pretreatment condition. There were no issues with feeding intolerance or recurrence of cyanosis during nasal feeding. The pulmonary symptoms diminished, and the infant experienced weight gain. The infant was discharged following a hospital stay of 17 days.

### 2.6. Genetic test results

Hematuria Tandem Mass Spectrometry: There were no apparent irregularities detected in the metabolic processes of amino acids and organic acids.

Duchenne muscular dystrophy (multiplex ligation-dependent probe amplification technique) detection of pseudo-hypertrophic muscular dystrophy: The Duchenne muscular dystrophy gene of the male infant with an X chromosome did not show any significant deletion or duplication of a large fragment in the exon.

**Figure 1.**
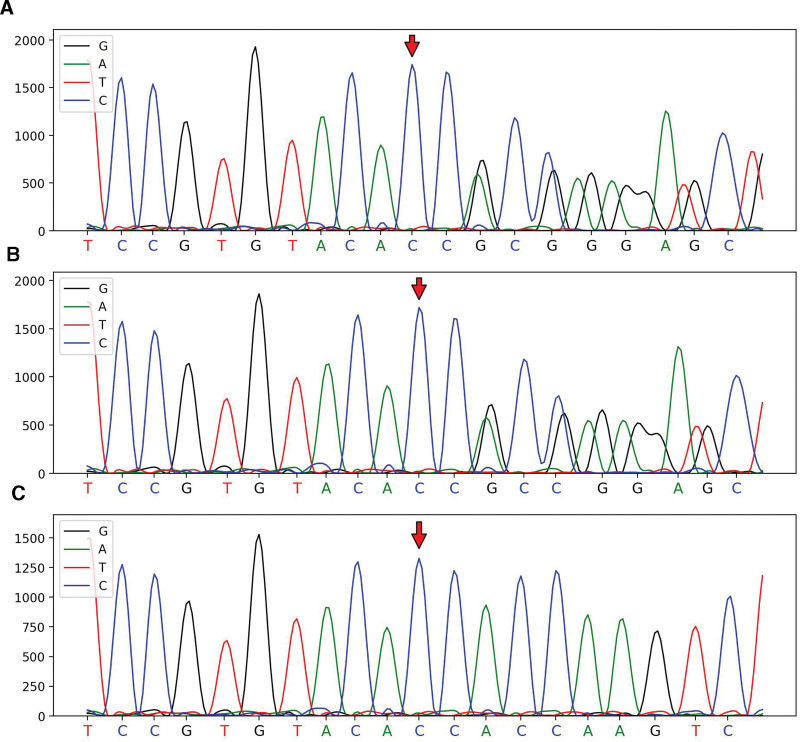
Genealogy verification map of first-generation sequencing. Validation sites: RR1,c.9661_9671 del, chromosomal location: chr19:39006831. (A) Proband, Heterozygous mutation at chr19:39006831. (B) Mother, Heterozygous mutation at chr19:39006831. (C) Father, wild-type at chr19:39006831.

Chromosomal Abnormality Detection Report: The sample did not exhibit any aneuploidy or identifiable and clearly defined genomic copy number variations larger than 100 Kb. Whole exon gene result: The phenotype aligns with the presence of a pathogenic or potentially pathogenic mutation that is associated with the RRR1 gene. The disease connected to this gene is CCD. The proband identified a potential pathogenic mutation in the RRR1 gene, and their observed symptoms match those typically seen in this disease (Fig. 1).

### 2.7. Discharge diagnosis

CCD (RYR1 gene mutation); Severe pneumonia; Influenza A virus infection; Type II respiratory failure; Obstructive sleep apnea syndrome; Myocardial damage; Liver function damage; Patent fossa ovalis; Gastroesophageal reflux.

## 3. Discussion

The infant experienced recurring foaming at the mouth, difficulty in feeding, slow weight gain, and a rash all over the body. A CT scan revealed pneumonia, despite the infant receiving anti-infection treatment at another hospital. Initially, we suspected milk protein allergy and gastroesophageal reflux aspiration as the common cause of recurrent pneumonia. We also considered the possibility of genetic metabolic disorders. As a result, we discontinued regular milk powder feeding and switched to a specialized milk powder with deep hydrolyzed protein. The infant underwent hematuria amino acid and organic acid tandem mass spectrometry testing. To prevent aspiration, we implemented nasal feeding and gradually increased the amount of food given. With the replacement of milk powder and symptomatic treatment, the clinical symptoms and signs gradually improved, allowing for a transition to oral feeding.

However, a few days later, the infant experienced excessive expectoration, leading to an electronic bronchoscopy. The bronchoscopy revealed relaxed pharyngeal muscles, retraction of the tongue root, inflammation of the tracheobronchial intima, and milk in the airway, indicating gastroesophageal reflux. We identified the loose pharyngeal muscle and retracted tongue root as causes of the uncoordinated swallowing function, leading to recurrent aspiration pneumonia along with gastroesophageal reflux. Additionally, the infant exhibited decreased muscle tension and strength in all 4 limbs, as well as poor head control. Elevated creatine kinase levels in the biochemistry test raised suspicion of muscular dystrophy or another muscular-related genetic disease. Subsequent specific tests for muscular dystrophy and second-generation sequencing of chromosome and whole exon genes were conducted.

The final report from the whole exon gene sequencing revealed a mutation in the RYR1 gene, leading to a diagnosis of CCD based on the clinical symptoms and signs. The infant mother was identified as the carrier of a heterozygous mutation in the RYR1 gene. CCD can manifest as either a dominant or recessive genetic disorder, and the identified heterozygous mutation aligns with the dominant inheritance pattern of the disease. CCD is more prevalent among infants than neonates. Infants with CCD, also referred to as “floppy baby,” experience slow motor development. A case in China reported similar symptoms in a 4-month-old boy, including recurrent respiratory symptoms, coughing, dependence on oxygen inhalation, reduced muscle strength and tension throughout the body, and delayed motor development. Initially, the boy was misdiagnosed with bronchopulmonary dysplasia and cerebral dysplasia.^[7]^ The presence of foaming at the mouth led to a pneumonia diagnosis, followed by a notable upper airway obstruction with a distinct triple concave sign, which was mistakenly attributed to softening of the thyroid cartilage. Consequently, it is crucial to promptly conduct bronchoscopy and genetic testing for infants experiencing recurrent pneumonia and hypotonia. Another report indicated that hip joint dislocation was the initial symptom in some infants, accompanied by a lack of apparent muscle strength and reduced muscle tension. As the disease progressed, walking difficulty worsened gradually, developmental delay occurred, facial muscle weakness emerged, scoliosis developed, and various degrees of reduced proximal muscle tension, muscle strength, and muscle volume were observed. Only a slight increase in creatine kinase levels was detected, and no abnormalities were found in the electromyography.^[8]^ These cases serve as reminders for clinicians to closely monitor the condition, conduct thorough physical examinations, and actively search for the underlying cause to avoid misdiagnosis. Previously, the identification of individuals suffering from CCD relied heavily on electromyography, creatine kinase, and analysis of muscle tissue. However, advancements in molecular genetic technology have introduced gene sequencing as a valuable aid in the clinical diagnosis of CCD. Through genetic testing of the infant, the specific gene responsible for the condition was identified, aligning with the observed clinical symptoms and signs. As a result, the diagnosis was confirmed. However, the parents declined further electromyography and muscle biopsy procedures. Currently, there is no specific treatment plan available for CCD. However, early rehabilitation training can be beneficial for children with CCD as it can ensure timely correction of deformities, maximize the development of the motor system, restore normal motor function, and improve the overall prognosis. Despite the infant weak muscles and uncoordinated swallowing, continuous nasogastric feeding can be employed to prevent choking and the aspiration of milk, thus reducing the risk of recurrent lower respiratory tract infections. Further follow-up is necessary to assess the long-term outlook for such children. The parents were educated on the relevant nursing knowledge regarding nasogastric feeding and post-treatment measures to address infant choking. It is recommended that the parents engage oral function and muscle rehabilitation training for the infant and regularly attend checkups to monitor the child growth and development through child health care services.

## 4. Conclusion

Initial symptoms of CCD may manifest as non-muscle or joint-related issues. During clinical diagnosis and treatment, medical professionals should be alert when encountering patients with recurrent pneumonia and low muscle tension, as it could indicate the presence of the disease. It is crucial to identify the underlying cause to prevent misdiagnosis and improper treatment. The utilization of next-generation gene sequencing has significantly aided in the accurate diagnosis of this condition.

## Acknowledgments

We are particularly grateful to all the people who have given us help on our article.

## Author contributions

**Conceptualization:** Qiu-yue Zhang, Xuan Xu.

**Formal analysis:** Qiu-yue Zhang, Lu Bai.

**Data curation:** Yang-yan Yin, Lu Bai.

**Writing – original draft:** Qiu-yue Zhang.

**Writing – review & editing:** Yang-yan Yin, Lu Bai, Xuan Xu.
